# Authors' Reply

**DOI:** 10.1371/journal.pgen.0020208

**Published:** 2006-11-24

**Authors:** Erik van Nimwegen, Mihaela Zavolan

## Authors' Reply

That splice variation at tandem acceptor sites is frequent has been reported by several groups, including Zavolan et al. [1], Sugnet et al. [2], and Hiller et al. [3], and is uncontroversial. It is to be expected that at least some of these variations will affect protein function, and this is also beyond dispute, in spite of suggestions to the contrary in the letter of Hiller et al. [4]. The questions that are under discussion concern the mechanism that brings about these splice variations and their “functional consequences” or “role in biological functions.” The rather vague formulation of these questions has, in our opinion, given rise to much misunderstanding. Therefore, to be concrete, we list what we believe are the main relevant questions. (1) Why are these splice variations so common? By what mechanism are they brought about? (2) To what extent is the introduction of these variations controlled and regulated by the cell? (3) What fraction of these variations is neutral and what fraction affects protein function? (4) To what extent are the non-neutral variations deleterious and to what extent are they beneficial?

With respect to the first question, we have shown [5] that one need not invoke a complicated mechanism for introducing these variations, but that a simple model of stochastic binding of the spliceosome to competing splice sites, in combination with nonsense-mediated decay, can fully explain the abundance of these variations. Moreover, this model accurately predicts the relative frequencies of all small length variations, not only at acceptor but also at donor splice sites. As Hiller et al. also stress in their letter, there is little doubt that thermodynamic fluctuations, i.e., noise, play a role in splice-site selection. The combination of these facts suggests to us that thermodynamic noise is responsible for introducing a large fraction of the observed alternative splicing events at tandem acceptors.

With respect to the second question, if the introduction of splice variation at NAGNAG acceptors were highly controlled by the cell, then one would not expect that they could be predicted from the local sequence at the splice site only. The fact that our same simple model successfully predicts which NAGNAG acceptors show splice variation and which do not suggests that at least a substantial fraction of all such splice variations are not tightly controlled by the cell. We agree with Hiller et al. that our model cannot explain the observed cases of variation in the relative proportion of the alternative splice forms across different tissues. We disagree, however, that this invalidates our model for these NAGNAGs. Just as different point mutations occur at different rates in different cellular states and sequence contexts, so may the relative probabilities with which the spliceosome binds to competing splice sites depend on details of the kinetics that may vary between tissues. It remains to be determined if the cells are able to actively regulate kinetic details so as to specifically regulate alternative splicing at tandem acceptor sites. In fact, we feel that one of the main uses of our model is to provide a baseline expectation under simple thermodynamic noise, allowing one to more effectively identify interesting cases that deviate significantly from this behavior.

With respect to questions 3 and 4, it is of course to be expected that some of the variations affect protein function. Indeed, Hiller et al. [3] have provided several lines of evidence that indicate a bias toward alternative NAGNAG acceptors that minimize the effect on the proteins. We agree with Hiller et al. that this strongly suggests that, at least in some cases, the effects of NAGNAG variations are deleterious and that selection acts to avoid them. We strongly disagree, however, that this argues against noise being responsible for *introducing* these variations. By the same reasoning one could argue that point mutations are not introduced by noise because one observes negative selection against certain single point mutants. Rather, the observed selection against NAGNAG motifs in locations where splice variation would deleteriously affect protein function suggests that the splice variation at NAGNAG acceptors is not under tight control of the cell, and supports the idea that these variations are mostly the result of uncontrollable noise. Finally, the fact that some variations deleteriously affect protein function does not imply that all these variations play a “role in biological function.” In many cases some amount of deleterious variation might just be tolerated.

How frequent are cases in which variations are beneficial for the cell, i.e., in which the cell uses both functionally different forms? We agree with Hiller et al. that such cases remain to be identified, but do not agree that the problem lies with the general difficulty of showing signs of positive selection. Positive selection is typically used to refer to cases where selection has favored *change* at particular positions. In contrast, to show that NAGNAG variations are beneficial, one would need to show only that there is clear selection for conserving the tandem acceptor property of variant NAGNAGs. This was in fact precisely the purpose of our test that compared the conservation of variant NAGNAG acceptors with that of invariant NAGNAG acceptors. Hiller et al. call this test “probably biased” due to a substantial fraction of NAGGAG tandem acceptors in which the GAG is part of the “conserved exon.” The point that we may not have stressed enough [5], and that is apparently not appreciated by Hiller et al., is that if there is selection for maintaining a NAGNAG acceptor that supports splice variation, then *both* AG dinucleotides need necessarily to remain conserved. This selection pressure is stronger even than the selection pressure on NAGs that are part of the exon, where selection will chiefly operate at the level of their coding potential, often allowing for neutral mutation of the AG dinucleotide. Thus, NAGNAGs at invariant acceptors must necessarily be under less selection to conserve both AG dinucleotides than beneficial variant NAGNAGs. If a substantial proportion of the variant NAGNAGs were under selection for their tandem acceptor property, then we would expect to see their NAGNAG property more often conserved than for invariant NAGNAGs. Since we do not observe this, we conclude that the fraction of NAGNAGs under selection for retaining their tandem acceptor function cannot be very large. Finally, Hiller et al. discuss the conservation test that they performed [3] and mention the conservation statistics obtained more recently by Akerman and Mandel-Gutfreund [6]. In Text S1 we discuss our interpretation of both these conservation tests.

## Supporting Information

Text S1. Conservation Patterns at NAGNAG Acceptor Sites (92 KB PDF)
