# Supplementary material for: Authors' Reply
Source: PLoS Genet. 2006 Nov 24;2(11):e208. doi: 10.1371/journal.pgen.0020208 (PMC1657061; doi:10.1371/journal.pgen.0020208)
Supplement: Text S1. — (92 KB PDF) [file pgen.0020208.sd001.pdf]

Supporting Material

Response to: Alternative splicing at NAGNAG acceptors: Simply noise or noise and more?

Erik van Nimwegen and Mihaela Zavolan
Division of Bioinformatics, Biozentrum, University of Basel,
Klingelbergstrasse 50-70, CH-4056 Basel, Switzerland
email: erik.vannimwegen@unibas.ch, mihaela.zavolan@unibas.ch

In their letter Hiller et al. mention the conservation test that was performed in [1] and the more recent results of Akerman and Mandel-Gutfreund [2]. To explain our interpretation of these results we first briefly describe our rough hypothesis for the origin of the bulk of NAGNAG splice variations.

We believe that in general the splicing machinery splices invariantly at the first AG that follows the polypyrimidine tract. This is supported by the fact that the large majority of invariant NAGNAGs splice indeed at the first NAG. Variant NAGNAGs can occur when, due to a combination of selection on the protein level and neutral evolution, a second NAG is introduced immediately downstream of the splice site. Especially when the major splice site undergoes some mutations that weaken its affinity for the spliceosome this may lead to a situation in which the spliceosome will sometimes 'slip' beyond the first NAG and splice at the second (exonic) NAG. In cases where this variation is not strongly deleterious, this 'slippery' splice site may persist for a significant amount of evolutionary time. In short, we hypothesize that most variant NAGNAGs have their major splice site at the first NAG. Under this hypothesis these NAGNAGs should show roughly the same conservation as invariant splice sites that splice at the first NAG. In contrast, invariant NAGNAGs that always splice at the second NAG would be expected to have a lower amount of selection on the first (always intronic) NAG.

In [1] Hiller et al. took variant NAGNAGs, i.e. those that show splice variation, and classified them as 'intronic' (extra NAG in the intron) or exonic (extra NAG in the exon) based on the RefSeq annotation of the transcript. For intronic NAGNAGs they observed a somewhat higher level of conservation of the A and G nucleotides at positions -5 and -4 than the A and G nucleotides at -5 and -4 from transcripts that do not have an AG dinucleotide at those positions. Such enhanced conservation was not observed for the class of exonic NAGNAGs.

We believe that these observations are precisely consistent with our hypothesis above: for many of these 'intronic' variant NAGNAGs the major splice site is really at the first NAG, but the RefSeq sequence happens to contain the minor form which splices at the second NAG. For these intronic NAGNAGs there will be a strong selection on maintaining the major splice site, i.e. the A and G at positions -5/-4. In contrast, the 'exonic' NAGNAGs have their splice site (almost always correctly) assigned to the first NAG, and these will not show more conservation than invariant NAGNAGs that splice at the first NAG.

We can in fact validate this explanation using our splice site selection model [3]. Our model predicts that we can distinguish NAGNAGs in which the first NAG is the major splice site, from those in which the second NAG is the major splice site, by the likelihoods of the sequences around the two putative acceptors for the acceptor site weigh matrix (ASWM). If the first NAG has much higher likelihood than the second we predict it to be the major splice site. Conversely, if the second NAG has much higher likelihood than the first we predict the second NAG to be the major splice site.

We collected all NAGNAG acceptors from our data [3] and calculated, for each, the difference in log-

likelihood of the two putative acceptor sites for the ASWM. We then calculated conservation statistics of the NAGNAGs as a function of this log-likelihood difference, as shown in figure 1. The horizontal axis shows

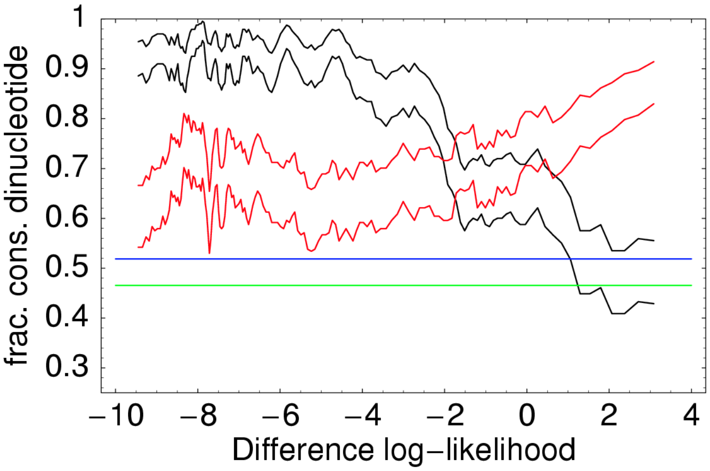

Figure 1: Fraction of AG dinucleotides in NAGNAG sites that are conserved between human and mouse as a function of the log-likelihood difference of the acceptor splice site weight matrix scores of the second and first AG splice site. The black lines show the fraction of times the first AG of the NAGNAG site is conserved plus and minus two standard deviations. The red lines show the fraction of times the second AG of the NAGNAG site is conserved plus and minus two standard deviations. The blue and green lines show the fraction of conserved intronic dinucleotides upstream of non-NAGNAG acceptors, i.e. the dinucleotides at position -5 and -4 in acceptors of the form NAHNAG (H means no G) and NBGNAG (B means no A) respectively.

the difference in log-likelihood of the second and first NAG splice site for the ASWM. That is, on the left are NAGNAGs that are predicted to have the major splice site at the first NAG, on the right those NAGNAGs that are predicted to have the major splice site at the second NAG, and around zero the NAGNAGs that are predicted to show splice variation. In black we show the fraction of NAGNAGs for which the first AG dinucleotide is conserved, and in red we show the fraction of NAGNAGs for which the second AG dinucleotide is conserved, both as a function of the log-likelihood difference. To compare these conservation profile with the conservation of intronic dinucleotides in non-NAGNAG acceptors the blue and green lines show the fraction of conserved dinucleotides at positions -5 and -4 of acceptors of the form NAHNAG and NBGNAG respectively.

First, notice that by far the strongest conservation is observed for the AG dinucleotides that are predicted to be the major splice site, i.e. the first NAG (black lines) on the left side of the figure and the second NAG (red lines) on the right side of the figure. For NAGNAGs predicted to splice at the first NAG (left hand side) we see that the second NAG (red lines) is more often conserved than intronic dinucleotides (blue and green lines). This is consistent with the fact that these AGs are part of the exon and are under selection for their protein coding potential. In contrast, we see that, as predicted, the NAGNAGs that are predicted to have the second NAG as major splice site (on the right of the figure) show no more conservation in the first NAG (black lines) than the dinucleotides at the same position in non-NAGNAG acceptors.

Very recently Akerman and Mandel-Gutfreund [2] have reported that the 30 bps upstream of variant NAGNAGs are more conserved on average than those upstream of invariant NAGNAGs. We have also attempted to compare these results with our own conservation tests. This task is complicated by the fact that some of the details of the data selection in [2] are not clearly described. It does appear, however, that the sets of NAGNAGs whose flanking intron conservation pattern were studied by Akerman and Mandel-Gutfreund were themselves selected based on conservation of the alternative splicing between human and

mouse. We consider it generally problematic to compare conservation statistics between data sets that were themselves selected based on conservation criteria since it is typically very hard to avoid the introduction of biases in such procedures. Therefore, we attempted to reproduce the results of [2] on our FANTOM3 dataset of variant and invariant NAGNAGs, in which we selected and classified the splice sites based only on the observed splice variation in mouse.

As we have done in our paper [3], we consider three classes of NAGNAG sites: those that always splice at the first NAG, those that always splice at the second NAG, and those showing splice variation. We obtained human/mouse conservation profiles for the 30 intronic nucleotides upstream of the splice site for all three classes, in analogy to the conservation profiles obtained by Akerman et al. [2]. We find that the conservation profile of invariant NAGNAGs that splice at the first NAG is statistically indistinguishable from the conservation profile of invariant NAGNAGs that splice at the second NAG. For variant NAGNAGs we produced two different profiles: one by considering the first NAG the major splice site, and one by considering the second NAG the major splice site (note that the conservation profiles obtained this way are identical up to a horizontal shift of 3 bp). We find that the first conservation profile, in which we consider the first NAG the major splice site, is statistically indistinguishable from the conservation profiles of the invariant NAGs. This is again consistent with our hypothesis that most variant NAGNAGs have their major splice site at the first NAG. In contrast, the profile obtained when the second NAG is considered the major splice site is shifted 3bps to the left and, since the conservation drops as one moves away from the splice site (as also observed by Akerman and Mandel-Gutfreund), this leads to an apparent increase of conservation for this profile. Although we cannot be sure, since details are missing in the paper, we suspect that Akerman and Mandel-Gutfreund may have chosen the second NAG in variant NAGNAGs as the major splice site, and in this way obtained a conservation profile for variant NAGNAGs that is effectively shifted to the left by 3 bps compared to the profiles of invariant NAGNAGs. Thus, we hypothesize that the results of Akerman and Mandel-Gutfreund may be due to a combination of bias caused by selecting data sets based on conservation criteria and erroneously classifying the second NAG as the major splice site, just as in the conservation test of Hiller et al.

References

[1] Hiller M, Huse K, Szafranski K, Jahn N, Hampe J, et al. (2004) Widespread occurrence of alternative splicing at NAGNAG acceptors contributes to proteome plasticity. Nat Genet 36.

[2] Akerman M, Mandel-Gutfreund Y (2006) Alternative splicing regulation at tandem 3' splice sites. Nucl Acids Res 34:23–31.

[3] Chern TM, van Nimwegen E, Kai C, Kawai J, Carninci P, et al. (2006) A simple physical model predicts small exon-length variations. PLoS Genet 2:e45.
